# Visual rehabilitation after LASIK complication: flap amputation, topo-guided surgery, and phacoemulsification

**DOI:** 10.5935/0004-2749.2023-0221

**Published:** 2024-03-27

**Authors:** Frederico França Marques, Daniel Filipe Oliveira Rabelo, Daniela Meira Villano Marques, Glauco Sérgio Avelino de Aquino, Daniel Diniz da Gama, Bernardo Kaplan Moscovici

**Affiliations:** 1 Department of Ophthalmology and Visual Sciences, Universidade Federal de São Paulo, São Paulo, SP, Brazil; 2 Marques Eye Institute Oftalmologia, São Paulo, SP, Brazil; 3 Department of Ophthalmology, Hospital Oftalmológico Visão Laser, Santos, SP, Brazil

**Keywords:** Refractive surgical procedures, Surgical flap/surgery, Keratomileusis laser In situ/methods, Biometry, Corneal topography, Lasers, Excimer/adverse effects, Dipoplia/etiologia, Visual acuity, Humans, Case reports

## Abstract

We present a case of a patient complaining of monocular diplopia due to a decentered ablation after LASIK. The patient underwent a wavefront-guided retreatment, which resulted in an epithelial ingrowth complication. Additionally, the patient developed cataract, with cataract surgery requiring reliable biometric measurements. Therefore, we opted for corneal treatment and corneal surface regularization. Although we attempted to lift the flap and wash the interface initially, the procedure proved unsuccessful, thereby necessitating immediate flap amputation. Once the corneal surface was regularized in the seventh postoperative month, transepithelial photorefractive keratectomy was successfully performed to homogenize the ocular surface, thereby significantly improving the patient’s corrected visual acuity and resolving monocular diplopia. The surface and corneal curvature stabilized by the fifth month after the procedure. Phacoemulsification was then performed along with the implantation of a toric monofocal lens, which was selected using an appropriate formula, resulting in an excellent uncorrected visual acuity.

## INTRODUCTION

Refractive procedures are generally safe, but they are associated with some risks. The risk rate reported for refractive procedures is <2%. LASIK-related postoperative complications include those related to the interface, such as fluid or cell accumulation. This accumulation promotes inflammatory and infectious processes, including epithelial growth and flap necrosis^(1^-^10)^.

We here present the treatment of a patient who developed monocular diplopia due to decentered ablation, interface epithelialization, and flap edge necrosis combined with cataract after an unsuccessful LASIK.

## CASE REPORT

A 55-year-old man presented to our service in 2016 complaining of monocular diplopia in the right eye (OD), which persisted since 2002 after the patient underwent LASIK in both eyes to correct myopic astigmatism. The patient reported that the treatment was not perfectly centralized. Wavefront-guided enhancement was performed 2 months later with no success.

**Figure 1:**
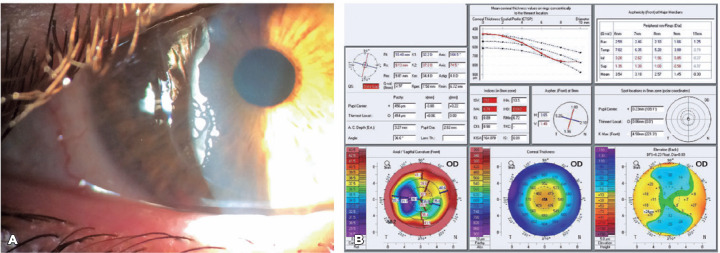
(A). Slit-lamp photopography of OD showing epithelial ingrowth and flap edge necrosis. (B). Scheimpflug Tomography of OD showing irregular astigmatism before surgery.

The corrected distance visual acuity (CDVA) was 20/60 for his right eye (OD) (+4.00 -2.00 150) and 20/20 in the left eye (OS). During slit-lamp examination, epithelial growth was noted in the OD temporal interface (Figure 1A).

Pentacam corneal tomography (Oculus, Germany) was performed in both eyes. This procedure revealed OD with a flat and irregular cornea with astigmatism ranging from 28D to 38D at the pupil area, which explains the development of monocular diplopia (Figure 1B).

On discussing the procedure for removing epithelial growth with the patient, the patient preferred to wait. Five years later (2021), he returned with a worsening visual acuity (VA) in the OD because of the presence of a subcapsular cataract along with monocular diplopia. Considering that phacoemulsification was required in the OD, we first addressed the cornea to regularize its surface and obtain reliable biometry. We were aware that we may have to amputate the lamella if any extremely friable tissue was found.

During surgery, we aimed to lift the lamella and remove the epithelial cells. However, the lamella was extremely friable and irregular. Therefore, the amputation of the lamella was planned, followed by the application of 0.02% mitomycin for 2 min and exhaustive washing for preventing haze development.

**Figure 2:**
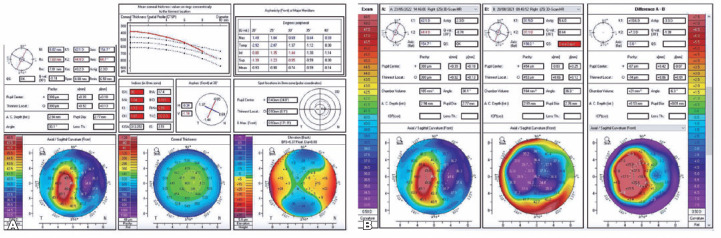
(A). Scheimpflug tomography of OD showing irregular astigmatism after our surgical intervention (flap amputation). (B). Differential Scheimpflug tomography of OD before and after flap amputation.

After 9 months, the patient presented a CDVA of 20/30 with a rigid contact lens and stable topography (Figure 2A and B).

**Figure 3:**
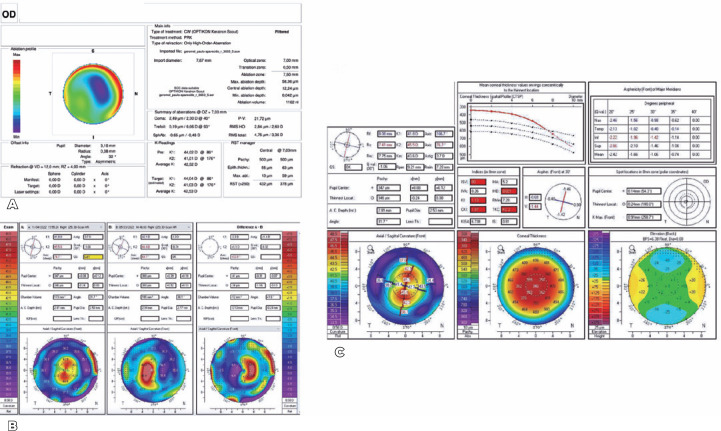
(A). Surgical topography (corneal wavefront)-guided surgery with Amaris 1050. (B). Scheimpflug tomography of OD showing regular astigmatism, after the topography-guided surgery. (C). Differential Scheimpflug tomography of OD before and after topography-guided surgery demonstrating applanation of steepest region and the flattening of the steepest region.

In June 2022, transepithelial photorefractive keratectomy (TransPRK) topography-guided surgery was performed using the Amaris 1050 platform (Schwind, Kleinotheim, Germany) (Figure 3A) to regularize the corneal surface. At the second and third months after operation, the patient’s CDVA remained at 20/25 with -3.50 DS -3.50 DC 150 with a topographic central symmetrical astigmatism of 3.7D, consistent with his manifest refraction. For the first time, the patient experienced a huge improvement in monocular diplopia because of the central regularity of the cornea (Figure 3B and C).

We then finally decided to resolve the cataract. Biometry was performed comparing several formulas such as Haigis L, Barrett True K, and Double K. Despite the patient being initially subjected to laser ablation for myopia, all formulas were used with a hyperopic correction because of the final prolate aspect of the cornea evidenced through topography.

**Figure 4:**
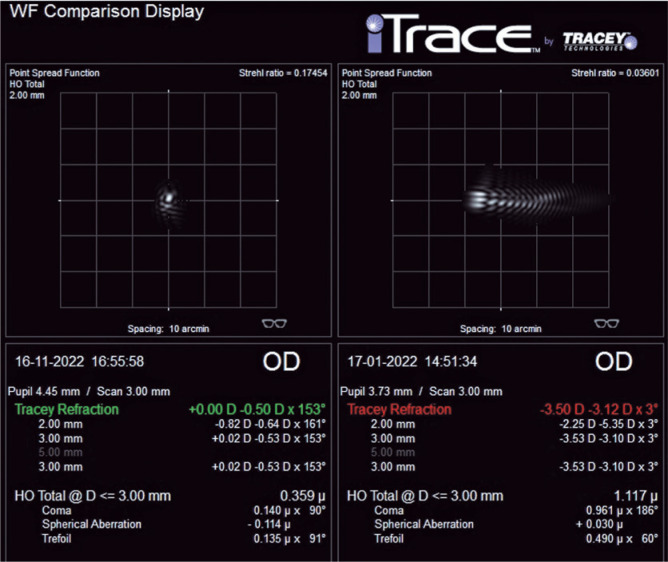
iTrace (high-order measurement) before and after treatments indicating improvement of the point spread function (PSF).

Therefore, in the fifth month after TransPRK, the patient was subjected to an uneventful phacoemulsification with implantation of a toric monofocal IOL, thereby resulting in an uncorrected VA (UCVA) of 20/20p with minimal monocular diplopia and comfortable binocular VA. Aberrometry using the point spread function (high-order) was used to confirm improvement after all treatments (Figure 4).

## DISCUSSION

Monocular diplopia is a rare postoperative complication of LASIK. Our patient presented with monocular diplopia in the OD due to decentered ablation, interface epithelialization, and flap edge necrosis combined with cataracts.

In patients developing monocular diplopia after LASIK, other potential causes of monocular diplopia, such as corneal irregularities or ocular misalignment, should be ruled out first.

Once the diagnosis is confirmed, treatment options vary depending on the etiology of the condition. In our patient, monocular diplopia was developed by two main causes: decentered ablation and epithelial ingrowth.

Decentered ablation is a rare complication that occurs nowadays because of an improvement in eye trackers. However, the incidence of the complication ranges between 0.2% and 4%, which results in high-order aberrations and low VA. Some authors have suggested that decentered or irregular flaps, head misalignment, or patient failure to fixate in the excimer laser-guiding light and high kappa angle are the leading causes of this complication^(1^-^3^,^9)^.

The gold standard for correcting decentered ablations is total wavefront-guided or corneal wavefront--guided treatments (topography-guided)^(4^,^5^,^9)^.

Several factors can contribute to epithelial ingrowth development, such as incomplete flap adherence, trauma, epithelial proliferation, greater ablations and hyperopic treatments, debris accumulation at the flap edge, and especially retreatments. The incidence of epithelial ingrowth after LASIK is 0.2%-2.0% in primary cases and 10%-20% in retreatments. Epithelial ingrowth is often asymptomatic or minimally symptomatic. However, it can lead to severe vision loss in some patients and require intervention^(1^-^6)^.

Epithelial ingrowth management can be challenging. As part of its typical treatment, the affected interface is mechanically dibridged. However, additional interventions such as alcohol, mitomycin C, fibrin glue, ND Yag laser, sutures, amniotic membrane grafts, or even flap amputation may be necessary for recurrent or refractory cases^(1^-^6)^.

In the present case, flap amputation was necessary because epithelial ingrowth was severe and irregular, which was followed by mitomycin C for preventing haze development. In cases with severe flap edge necrosis, closing the pathway for epithelial ingrowth is challenging, and using sutures and fibrin glue can be attempted. However, we could not achieve an acceptable outcome because of necrosis, as observed in some published cases. When amputation of the LASIK flap is decided, mitomycin C must be applied to avoid haze formation, and we must be prepared for irregular topography in the postoperative period^(6^-^8)^.

The patient presented a stable topography after the procedure was completed, but corneal irregularity persisted. Therefore, a TransPRK topography-guided procedure was performed. Topography-guided ablation allows to precisely correct corneal irregularities and can significantly improve VA, as observed in our case. Both waveguided and topography-guided treatments exhibit excellent results in treating irregular corneas. One among the two approaches should be chosen based on the surgeon’s expertise with the technology and availability^(4^,^5^,^9)^.

Because the cornea was irregular, some devices that perform wavefront-guided surgeries could not capture reliable images. Topography-guided surgeries aim to correct the cornea’s shape without considering the patient’s ametropia. Residual ametropia was not a concern in our patient because the patient had to undergo cataract surgery after topographical regularization^(4^,^5^,^9)^.

In the present case, the formula used for biometry was essential in achieving the desired outcome. Several formulas were considered, but because of the final prolate aspect of the cornea observed through topography, all formulas were used with hyperopic correction^(10)^.

In conclusion, the present case revealed that even after some postoperative excimer laser complications, the patient may still achieve excellent functional vision through the application of correct treatments.

## Statistical analysis:

**Obtaining funding:**

**Administrative, technical, or material support supervision:** Frederico França Marques, Bernardo Kaplan Moscovici, Daniel Diniz da Gama. **Research group leadership:** Frederico França Marques, Bernardo Kaplan Moscovici.
